# Case Report: Deep cervical lymphovenous bypass for Parkinson’s disease

**DOI:** 10.3389/fnins.2025.1707554

**Published:** 2025-11-26

**Authors:** Feiyun Wang, Ruilei Guan, Guanyu Yang, Dongya Zhang, Zhengkai Li, Yan Wang, Hailong Bing, Gaiqing Yang, Meng Mao, Qinjun Chu

**Affiliations:** 1 Department of Microsurgery, Zhengzhou Central Hospital Affiliated to Zhengzhou University, Zhengzhou, Henan, China; 2 Department of Neurology, Zhengzhou Central Hospital Affiliated to Zhengzhou University, Zhengzhou, Henan, China; 3 Department of Anesthesiology and Perioperative Medicine, Zhengzhou Central Hospital Affiliated to Zhengzhou University, Zhengzhou, Henan, China

**Keywords:** Parkinson’s disease, deep cervical lymphovenous bypass, lymphaticdrainage, neurosurgery, case report

## Abstract

**Background:**

Parkinson’s Disease (PD) is a progressive neurodegenerative disorder characterized by dopaminergic neuronal loss and *α*-synuclein aggregation. Current treatments do not halt disease progression. Recent research highlights impaired cerebral lymphatic drainage in PD, suggesting a potential therapeutic target.

**Case presentation:**

We report a 56-year-old male with advanced PD who underwent deep cervical lymphovenous bypass (DCLB) surgery, a novel intervention designed to enhance lymphatic drainage by anastomosing the deep cervical lymphatic vessel to a vein. The patient had been diagnosed with PD in 2024 after experiencing progressive motor symptoms since 2019, with suboptimal response to conventional pharmacotherapy.

**Results:**

At three-month follow-up, significant improvements were observed: Movement Disorder Society–Unified Parkinson’s Disease Rating Scale decreased by 30.4% (from 23 to 16), Non-Motor Symptoms Scale decreased by 71.4% (from 77 to 22), and Parkinson’s Disease Questionnaire-39 decreased by 94.2% (from 52 to 3). Both motor symptoms (rigidity, bradykinesia) and non-motor symptoms (sleep disturbances, orthostatic dizziness) showed improvement.

**Conclusion:**

DCLB was technically feasible and was followed by symptomatic improvements in this single patient. The underlying mechanism for this clinical response remains unclear and was not investigated with biomarkers in this report. While these preliminary findings are hypothesis-generating, causality cannot be inferred from an individual case. Further controlled studies incorporating neuroimaging and fluid biomarkers are needed to explore potential mechanisms.

## Introduction

PD represents the second most prevalent neurodegenerative disorder globally, significantly affecting quality of life among elderly populations (Kalia et al., 2015; Tolosa et al., 2021; Bloem et al., 2021). Epidemiological evidence indicates that the incidence of PD has increased over the past three decades, with approximately 6 million older adults currently affected (Dorsey et al., 2018). The primary pathological hallmarks of this progressive neurological condition include dopaminergic neuronal loss in the substantia nigra pars compacta and the formation of Lewy bodies, wherein the aggregation of *α*-synuclein is considered a critical pathogenic factor (Tanner and Ostrem, 2024).

Despite existing therapeutic approaches, including pharmacological interventions and surgical procedures, which can partially alleviate symptoms, these methods have not been shown to halt disease progression (Tanner and Ostrem, 2024). Consequently, there is continued interest in developing new therapeutic strategies. In recent years, advances in neuroscientific research have focused on cerebral metabolic waste clearance mechanisms. The glymphatic and meningeal lymphatic systems have been proposed to play roles in maintaining cerebral metabolic homeostasis (Louveau et al., 2015; Bartlett et al., 2024; He et al., 2020).

**Figure 1:**
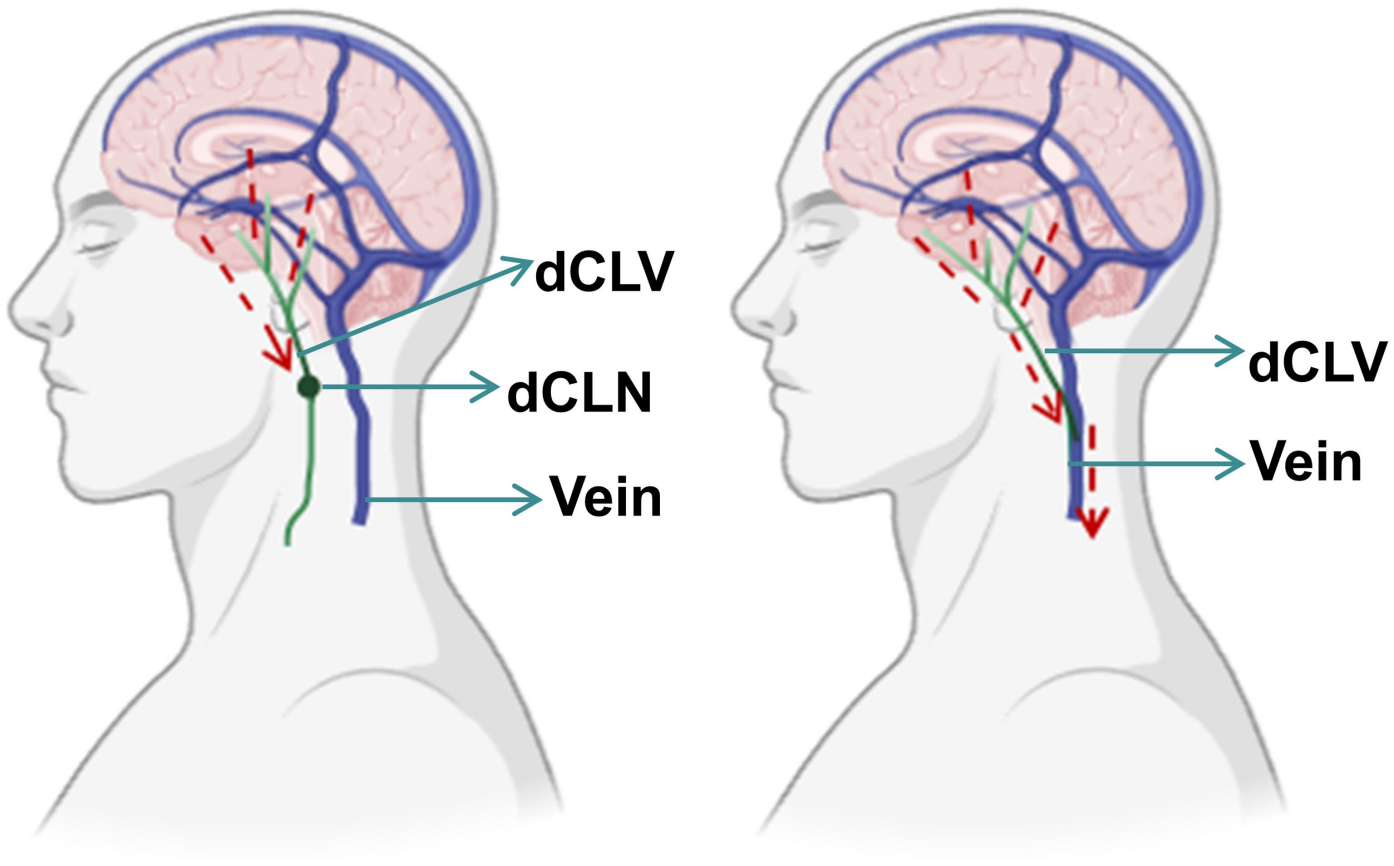
Schematic diagram of the deep cervical lymphovenous bypass procedure. dCLV, deep cervical lymphatic vessel; dCLN, deep cervical lymph node. This schematic was adapted from Yang et al. (2025).

In patients with PD, several studies have reported impaired lymphatic system function, which may contribute to reduced metabolic waste clearance (Dong et al., 2024; Liu et al., 2023; Visanji and Lang, 2021). In murine models, alterations of deep cervical lymphatic vessel (dCLV) function and blockade of meningeal lymphatic drainage have been associated with increased *α*-synuclein aggregation, activation of glial cells, and worsening of PD-like pathology (Zou et al., 2019). Clinical observations suggest that brain lymphatic drainage rates and cervical lymph node perfusion can be lower in PD patients compared to healthy controls (Ding et al., 2021). Notably, the conceptual framework of treating impaired cerebral clearance as a form of ‘cerebral lymphedema’ has recently been translated into clinical practice. Deep cervical lymphovenous anastomosis has been preliminarily investigated for Alzheimer’s disease (AD), with a recent systematic review identifying several case reports and a prospective cohort study suggesting potential short-term cognitive and behavioral improvements (Lahmar et al., 2025). One such cohort study of 26 AD patients found a significant improvement in Mini-Mental Status Examination scores one month after deep cervical lymphovenous anastomosis (Chen et al., 2025). Based on these findings, a surgical intervention has been proposed to enhance cerebral lymphatic system function through a DCLB procedure—specifically, anastomosing the deep cervical lymphatic vessel to the deep cervical vein (Figure 1). This approach is hypothesized to potentially influence symptom burden in PD. The present study is a proof-of-concept case report that aims to document the technical feasibility and short-term clinical outcomes of DCLB in a single patient with PD. It does not aim to provide direct evidence for the underlying mechanism, which remains to be investigated in future studies.

## Case description

A 56-year-old male patient presented with a 5-year history of progressive motor symptoms. The patient had no prior history of pesticide exposure, gas poisoning, habitual consumption of strong tea or coffee, or use of antipsychotic medications. There was no family history of genetic diseases or movement disorders. The patient’s clinical timeline began in 2019 when he experienced stiffness and progressive weakness in his left lower limb, initially treated as peripheral nerve disease without satisfactory results. In 2021, he developed lower back pain and bradykinesia, which was managed as lumbar disc herniation, also without improvement. In May 2024, he was formally diagnosed with PD at the First Affiliated Hospital of Henan University of Traditional Chinese Medicine and initiated treatment with levodopa-benserazide, entacapone, and carbidopa-levodopa with suboptimal response. In July 2024, pramipexole was added to his treatment regimen with initial improvement. However, he subsequently developed “on–off” fluctuations with prominent wearing-off effects, leading to progressive functional decline.

Upon admission to our institution in August 2024, comprehensive laboratory investigations including parathyroid hormone levels, antiphospholipid antibodies, tumor markers, antinuclear antibody panel, and thyroid function tests were unremarkable. Neuroimaging evaluation encompassing brain MRI, MRA, SWI, and DTI revealed white matter lesions (Fazekas grade 2), a dominant left vertebral artery, and reduced fiber tract density within the left inferior fronto-occipital fasciculus and corticospinal tract. FDG-PET metabolic imaging demonstrated reduced glucose metabolism in the lateral aspect of the right temporal lobe, medial aspects of both frontal lobes, and medial aspect of the left temporal lobe. Physical examination revealed primary motor symptoms including muscle rigidity (predominantly left-sided), bradykinesia, and postural instability with positive pull test. Non-motor manifestations included hyposmia, orthostatic dizziness, hyperhidrosis, anxiety-depression spectrum symptoms, sleep-onset insomnia, and restless legs syndrome. The patient experienced significant motor complications including wearing-off phenomena with “on” time reduced to 2–3 h and dystonia during “off” periods.

The diagnosis of PD was confirmed based on the Berg et al. (2015), with the patient meeting core criteria (bradykinesia plus rigidity) and two supportive criteria (clear beneficial response to dopaminergic therapy with >30% improvement on levodopa challenge test, and presence of motor fluctuations). The timeline of the patient’s clinical course and intervention is summarized in Table 1.

**Table 1:** Timeline of clinical course and intervention.

Date	Clinical event	Key Findings/interventions
2019	Symptom onset	Left lower limb stiffness and weakness
2021	Symptom progression	Development of bradykinesia and back pain
May 2024	PD diagnosis	Initiated dopaminergic therapy
July 2024	Treatment modification	Added pramipexole; developed motor fluctuations
August 2024	Pre-surgical assessment	MDS-UPDRS III: 23; NMSS: 77; PDQ-39: 52
August 2024	DCLB surgery	Successful lymphovenous anastomosis performed
Week 1 post-op	Early response	Subjective reduction in muscle stiffness
Month 1 post-op	Intermediate assessment	MDS-UPDRS III: 17; NMSS: 22; PDQ-39: 3
Month 3 post-op	Final assessment	MDS-UPDRS III: 16; NMSS: 22; PDQ-39: 3

DCLB, deep cervical lymphovenous bypass; MDS-UPDRS, Movement Disorder. Society–Unified Parkinson’s Disease Rating Scale; NMSS, Non-Motor Symptoms. Scale; PDQ, Parkinson’s Disease Questionnaire.

## Diagnostic assessment and therapeutic intervention

### Rationale for DCLB

The patient was managed with combination anti-Parkinsonian therapy including levodopa-carbidopa, pramipexole, entacapone, and carbidopa-levodopa-entacapone. However, persistent motor complications including wearing-off, dystonia, reduced “on” time, and diminished drug efficacy resulted in declining quality of life. Deep brain stimulation (DBS) was recommended but declined due to financial constraints. The decision to proceed with DCLB was made after careful consideration of the limited alternatives. The theoretical rationale for selecting this specific procedure over other lymphatic interventions was based on its proposed site of action. While non-invasive methods like manual lymphatic drainage primarily influence superficial vessels, DCLB targets the deep cervical lymphatics, which constitute a major efflux pathway for cerebral interstitial fluid and metabolic waste, as suggested by preclinical models (Bartlett et al., 2024; He et al., 2020). Thus, DCLB was hypothesized to potentially enhance central waste clearance more directly than peripheral techniques. In contrast to DBS, which modulates neural circuitry, DCLB represents a fundamentally different approach aimed at a putative pathophysiological mechanism in PD—impaired glymphatic and meningeal lymphatic drainage (Dong et al., 2024; Liu et al., 2023; Visanji and Lang, 2021).

Based on this initial experience, we propose that consideration of DCLB might be guided by the following factors. Potential eligibility criteria could include: (1) a diagnosis of advanced PD with significant disability despite optimized medical therapy; (2) the presence of troublesome motor fluctuations or non-motor symptoms contributing to poor quality of life; (3) not being an ideal candidate for or declining established advanced therapies like DBS; and (4) a comprehensive preoperative evaluation that confirms the absence of contraindications. Such contraindications would likely include significant medical comorbidities conferring high surgical risk, coagulopathy, active infection, or unfavorable cervical anatomy that would preclude safe microsurgical anastomosis.

The potential benefits discussed with the patient and family were explicitly framed as speculative and derived from this mechanistic hypothesis. These included the possibility of influencing a broad range of symptoms by improving waste clearance and the theoretical, yet unproven, potential to slow pathological progression. These considerations were balanced against the definite risks of an open cervical procedure, including infection, bleeding, injury to critical nerves (e.g., the spinal accessory nerve), lymphatic leakage, thrombosis, and anastomotic failure (Kukreja-Pandey et al., 2024). The unknown long-term efficacy, durability, and the technical challenge of reliably identifying suitable deep lymphatic vessels were also emphasized as significant uncertainties.

Furthermore, for any future trials, strict ethical considerations must be paramount. These include the imperative for a robust informed consent process that transparently communicates the highly experimental nature of the procedure, the absence of long-term efficacy and safety data, and the potential for unforeseen consequences. Study protocols should ideally incorporate independent data and safety monitoring boards.

After a comprehensive discussion of these theoretical benefits, concrete risks, the lack of established alternatives, and the explicit understanding of the procedure’s experimental nature, the patient and family provided informed consent to proceed with DCLB surgery.

### Surgical procedure

The DCLB procedure was performed under general anesthesia. The total operative time was 170 min, with an estimated blood loss of 20 mL. No intraoperative complications occurred. The surgery was conducted following ethics approval (ZXYY2024218). All microsurgical steps were performed using a surgical microscope at magnifications between 20x and 30x, adjusted for optimal visualization of the delicate structures.

**Figure 2:**
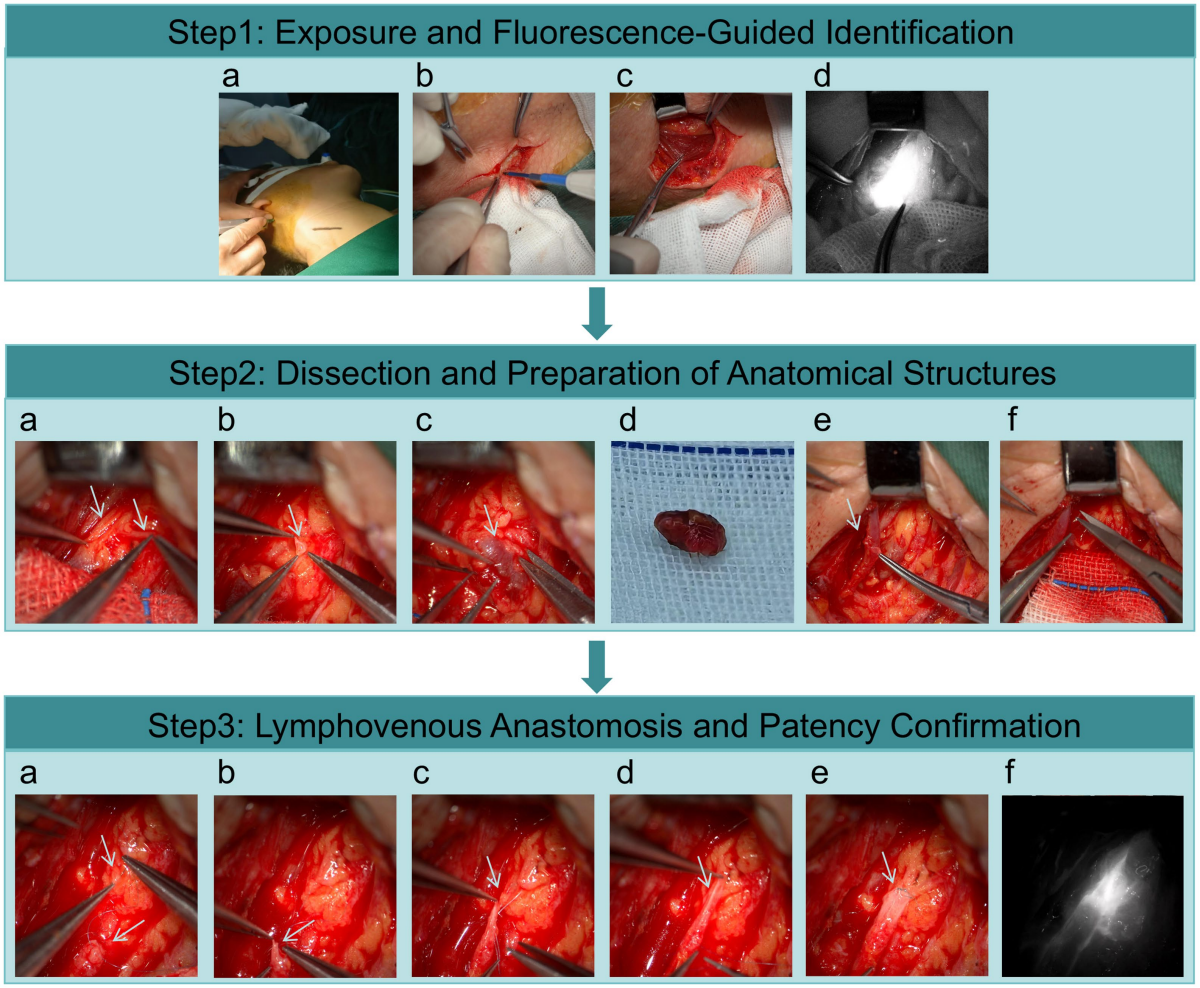
Surgical procedure for the deep cervical lymphovenous bypass. Step 1: exposure and fluorescence-guided identification. a: Subcutaneous injection of indocyanine green into the retroauricular (mastoid), mandibular angle, and occipital regions. A longitudinal incision was made along the posterior border of the sternocleidomastoid muscle. b: Incision of the skin and subcutaneous tissue. c: Dissection and identification of the sternocleidomastoid muscle, which was retracted medially. d: Fluorescence imaging revealing the deep cervical lymph nodes. Step 2: Dissection and Preparation of Anatomical Structures. a: Exposure of a recipient vein (indicated by the left white arrow) and an afferent lymphatic vessel (indicated by the right white arrow). b: Visualization of lymph fluid outflow from the lymphatic vessel. c: Intraoperative view of a deep cervical lymph node. d: Excision of the target lymph node. e: Dissection and preparation of a suitable deep cervical vein for anastomosis. f: The recipient vein was ligated distally and transected after complete mobilization, preparing for the lymphovenous anastomosis. Step 3: Lymphovenous Anastomosis and Patency Confirmation. a–d: Sequential views of the octopus-style (sleeve) anastomosis between the lymphatic vessel and the vein. e: Final appearance of the completed anastomosis. f: Indocyanine green fluorescence angiography confirming patency, with lymph flow into the venous system.

The surgical technique (Figure 2) involved: (1) Subcutaneous injection of indocyanine green at the mastoid, mandibular angle, and occipital regions for fluorescence-guided lymphatic visualization; (2) Longitudinal incision along the posterior border of the sternocleidomastoid muscle with careful dissection to identify deep cervical lymph nodes; (3) Under microscopic guidance, the dCLNs and their associated vessels were identified. The targeted dCLVs typically had external diameters ranging from 0.1 mm to 0.5 mm. The primary criteria for selecting a suitable lymphatic vessel for anastomosis were: (a) a pliable wall without significant fibrosis or inflammation; and (b) robust, spontaneous lymph flow from the transected end, indicative of good inflow. Afferent vessels leading into a lymph node were preferred; (4) A suitable recipient vein was meticulously dissected. The selection of the recipient vein was not predetermined but was based on achieving a size match with the prepared lymphatic vessel. Potential recipient veins included subcutaneous veins, small branches of the internal or external jugular veins, or veins accompanying the deep cervical artery, with diameters typically ranging from 1.0 mm to 3.0 mm; (5) An end-to-end anastomosis was performed under high magnification; (6) Patency was confirmed intraoperatively by observing the direct passage of indocyanine green fluorescence from the lymphatic vessel into the venous system. While intraoperative fluorescent angiography served as the primary patency check, future studies could incorporate postoperative assays of Parkinson’s disease-relevant biomarkers in blood (e.g., *α*-synuclein) as an indirect, systemic measure of the potential enhancement in central waste clearance.

**Figure 3:**
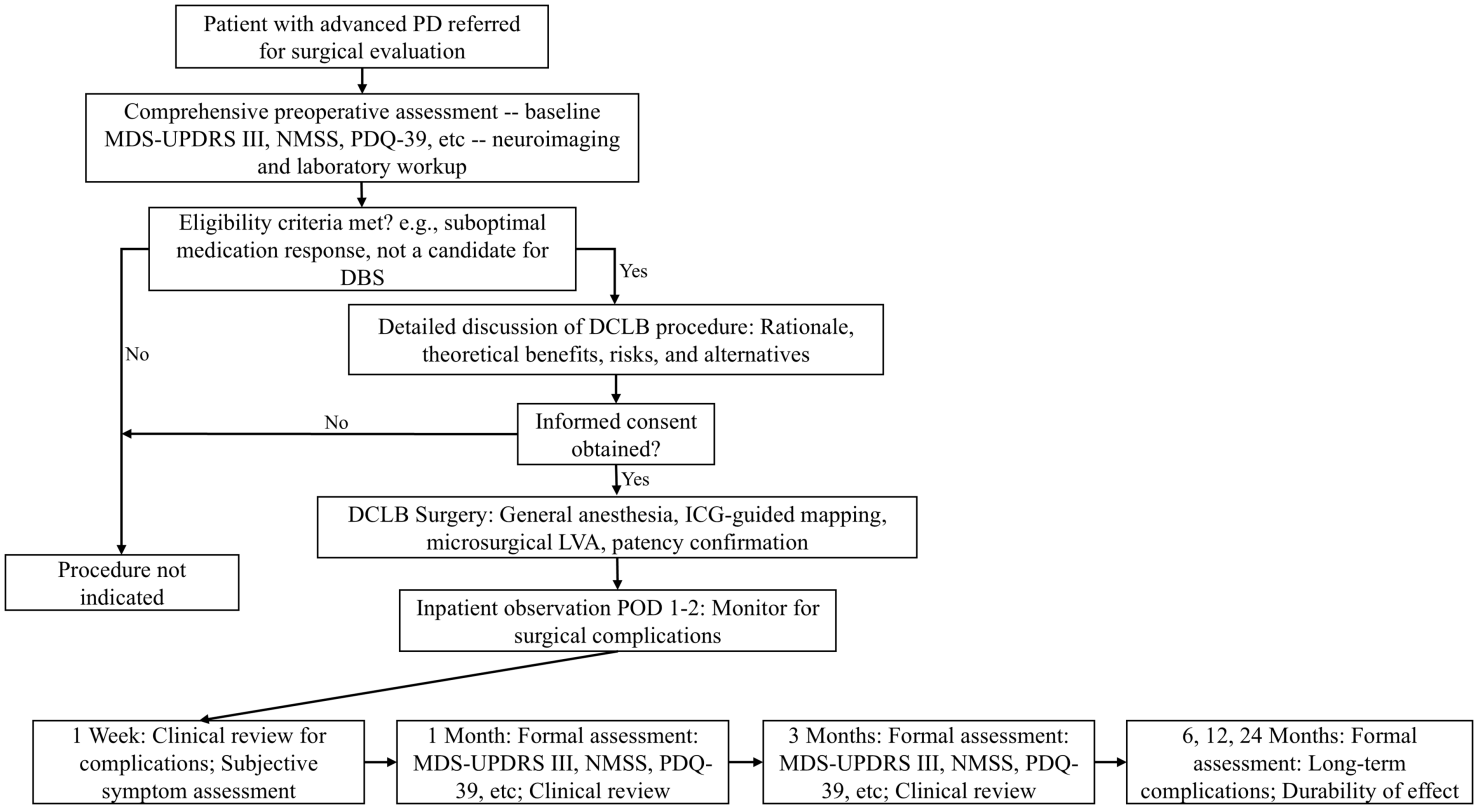
Perioperative workflow and postoperative monitoring protocol for deep cervical lymphovenous bypass (DCLB). This flowchart summarizes the key stages of patient management, from preoperative evaluation to long-term follow-up. PD, Parkinson’s Disease; MDS-UPDRS, Movement Disorder Society–Unified Parkinson’s Disease Rating Scale; NMSS, Non-Motor Symptoms Scale; PDQ-39, Parkinson’s Disease Questionnaire-39; DBS, deep brain stimulation; ICG, indocyanine green; IVA, lymphovenous anastomosis; POD, postoperative day.

The overall perioperative management strategy, from initial preoperative evaluation through to long-term follow-up, is summarized in Figure 3.

### Follow-up and outcomes

Throughout the three-month postoperative follow-up period, the patient’s anti-Parkinson’s medication regimen was maintained unchanged from the preoperative period.

#### One week post-operative

The patient reported subjective reduction in muscle stiffness with no surgical complications observed.

#### One month post-operative

Significant improvements were noted including alleviation of restless legs syndrome, improved ability to turn over in bed, enhanced postural balance (negative pull test), improved fine motor skills, and extension of “on” period from 2–3 h to 4–5 h. Assessment scale scores were as follows: MDS-UPDRS III decreased to 17 (26% reduction), NMSS decreased to 22 (71.4% reduction), and PDQ-39 decreased to 3 (94.2% reduction).

#### Three months post-operative

Additional improvements included reduced orthostatic dizziness, improved speech clarity and swallowing function. Scale scores remained stable with MDS-UPDRS III at 16 (30.4% reduction from baseline), NMSS at 22 (71.4% reduction maintained), and PDQ-39 at 3 (94.2% reduction maintained).

## Discussion

This case report describes the short-term clinical outcomes following DCLB in a single patient with advanced PD. The procedure was technically feasible and its performance coincided with substantial improvements across motor symptoms, non-motor symptoms, and quality-of-life metrics over a three-month follow-up period, as evidenced by pronounced reductions in MDS-UPDRS III, NMSS, and PDQ-39 scores.

Potential explanations for the observed clinical changes, which future studies should explore, include enhanced clearance of pathological proteins like *α*-synuclein and modulation of neuroinflammatory responses (Zou et al., 2019; Ding et al., 2021). The relatively more pronounced improvement in non-motor symptoms could be consistent with a rapid anti-inflammatory effect or greater plasticity in non-motor circuits, whereas motor deficits may be limited by irreversible dopaminergic loss (Wichmann et al., 2025; Lin et al., 2025; Zhu et al., 2025). These remain hypothetical considerations unsupported by our data.

Our preliminary findings align with emerging evidence suggesting that surgical enhancement of cervical lymphatic drainage may confer symptomatic benefits across neurological conditions. The most direct parallels are drawn from DCLB in AD. For instance, Chen et al. reported significant short-term cognitive improvement in AD patients after deep cervical lymphovenous anastomosis, mirroring the rapid benefits observed in our case (Chen et al., 2025). A systematic review by Lahmar et al. further supports this, noting a consistent trend of early postoperative improvement across multiple AD case reports (Lahmar et al., 2025). The broad symptomatic benefit pattern in our patient, particularly in non-motor domains, finds a parallel in AD studies where neuropsychiatric inventory scores improved. This is consistent with the hypothesis that enhanced clearance may impact diverse neural circuits. Further supporting a potential brain-wide effect, von Reibnitz et al. described significant functional improvements in a patient with a central lymphatic anomaly following deep cervical LVA, demonstrating that modulating cervical flow can influence brain function even in non-degenerative contexts (von Reibnitz et al., 2025). Collectively, these observations suggest that DCLB appears to be associated with short-term symptomatic improvements across conditions, though the definitive mechanisms and durability remain key unanswered questions.

Placing our patient’s outcomes in the context of established DBS therapies is instructive. The landmark INTREPID trial demonstrated a substantial 51% improvement in off-medication MDS-UPDRS III at one year with STN-DBS (Starr et al., 2025), compared to our more moderate 30.4% motor improvement at three months. However, the scale of non-motor and quality-of-life benefits after DCLB is striking. The 71.4% reduction in NMSS and 94.2% reduction in PDQ-39 in our case appear to exceed the typical non-motor benefits of conventional DBS, where quality-of-life gains often parallel motor improvements and may decline over time (Starr et al., 2025). This pattern also contrasts with a recent trial of directional DBS, which, while effective for motor symptoms, did not yield superior non-motor outcomes compared to omnidirectional stimulation (Kukreja-Pandey et al., 2024; Gharabaghi et al., 2024). Interestingly, the comprehensive benefit seen with DCLB finds a closer parallel in studies of adaptive DBS (aDBS). A multicenter trial showed that aDBS outperformed conventional DBS in activities of daily living, motor complications, quality of life, and medication reduction over one year (Li et al., 2025), suggesting that interventions dynamically interacting with pathophysiological states—whether via neural feedback or potentially enhanced clearance—may offer a broader therapeutic profile. These cross-study comparisons, while preliminary, suggest DCLB might engage a fundamentally different mechanism, leading to a rapid and pronounced benefit in non-motor domains that warrants further investigation.

While DCLB was well-tolerated here, its potential risks must be acknowledged. These include standard microsurgical risks such as infection, bleeding, injury to critical neck neurovascular structures (e.g., spinal accessory or vagus nerves), lymphatic leakage, thrombosis, and anastomotic failure (Lahmar et al., 2025). The long-term consequences of altering deep cervical lymphatic drainage are unknown, including the theoretical concern that diverting lymph flow could disrupt immune surveillance, potentially increasing infection susceptibility or altering neuroimmune responses—a hypothesis requiring future investigation (Lahmar et al., 2025).

The methodological limitations of this single-case report profoundly limit causal inference. The absence of a control group, blinding, or randomization, coupled with the lack of pre- and post-operative biomarker assessments (e.g., CSF, PET, or functional lymphatic imaging), means the observed improvements cannot be definitively attributed to enhanced waste clearance. Notably, the observed improvements cannot be definitively disentangled from potential placebo effects or regression to the mean, in addition to the influences of concurrent medications or natural symptom fluctuations. The short-term follow-up is an important limitation that precludes assessment of long-term safety, the durability of the observed benefits, and potential late complications. Ongoing monitoring is in progress, but future controlled studies with larger cohorts and longer follow-up are imperative.

Given the preliminary nature of our findings in a single patient, DCLB’s applicability to different PD demographics remains an open question. Pathophysiologically, impaired glymphatic/lymphatic function is not thought to be restricted by age or gender (Iliff et al., 2012). Therefore, if DCLB’s therapeutic mechanism is indeed augmented waste clearance, its potential benefit could extend to a broader PD population, provided patients are suitable surgical candidates. Key future considerations include general surgical risk (e.g., in elderly patients with comorbidities), anatomical variations, and the optimal disease stage for intervention—potentially earlier, before extensive neuronal loss. Ultimately, defining the precise patient profile for DCLB requires prospective, controlled trials with diverse cohorts.

This report is intentionally focused on establishing the short-term feasibility and safety of the DCLB procedure. We are conducting ongoing monitoring of this patient and plan to report the long-term outcomes in a subsequent publication dedicated solely to the durability, evolution, and stability of the treatment effect over an extended period, which will be critical for understanding its interaction with the natural history of PD.

### Patient perspective

The patient expressed significant satisfaction with the outcomes, stating: “The improvement in my quality of life has been remarkable. Before the surgery, I was struggling with basic daily activities and the unpredictability of my symptoms was causing significant distress to both me and my family. The reduction in stiffness and the extension of my “on” periods have allowed me to regain some independence. Most notably, the improvement in my sleep and reduction in dizziness have made a tremendous difference in my daily functioning. While I understand this is a new procedure and long-term outcomes are unknown, I am grateful for the opportunity to have participated in this innovative approach and hope it may help other patients in the future”.

## Conclusion

This case report describes the application of DCLB in a patient with PD. The procedure was technically feasible and its performance coincided with notable improvements in motor and non-motor symptoms and quality of life over a three-month period. While these preliminary findings are encouraging and support further investigation into the role of lymphatic dysfunction in PD, they cannot establish efficacy or causality. The therapeutic mechanism remains speculative. Rigorous prospective controlled trials with larger cohorts, longer follow-up, and incorporation of biomarker assessments are necessary to evaluate the safety, efficacy, and potential clinical utility of DCLB for PD.

## Data Availability

The original contributions presented in the study are included in the article/supplementary material, further inquiries can be directed to the corresponding authors.
